# Comprehensive Analysis of the Potential Toxicity of Magnetic Iron Oxide Nanoparticles for Medical Applications: Cellular Mechanisms and Systemic Effects

**DOI:** 10.3390/ijms252212013

**Published:** 2024-11-08

**Authors:** Julia Nowak-Jary, Beata Machnicka

**Affiliations:** Department of Biotechnology, Institute of Biological Sciences, University of Zielona Gora, Prof. Z. Szafrana 1, 65-516 Zielona Gora, Poland; b.machnicka@wnb.uz.zgora.pl

**Keywords:** magnetic nanoparticles, iron oxide nanoparticles, nanotoxicity, oxidative stress, nanomedicine, nanobiotechnology

## Abstract

Owing to recent advancements in nanotechnology, magnetic iron oxide nanoparticles (MNPs), particularly magnetite (Fe_3_O_4_) and maghemite (γ-Fe_2_O_3_), are currently widely employed in the field of medicine. These MNPs, characterized by their large specific surface area, potential for diverse functionalization, and magnetic properties, have found application in various medical domains, including tumor imaging (MRI), radiolabelling, internal radiotherapy, hyperthermia, gene therapy, drug delivery, and theranostics. However, ensuring the non-toxicity of MNPs when employed in medical practices is paramount. Thus, ongoing research endeavors are essential to comprehensively understand and address potential toxicological implications associated with their usage. This review aims to present the latest research and findings on assessing the potential toxicity of magnetic nanoparticles. It meticulously delineates the primary mechanisms of MNP toxicity at the cellular level, encompassing oxidative stress, genotoxic effects, disruption of the cytoskeleton, cell membrane perturbation, alterations in the cell cycle, dysregulation of gene expression, inflammatory response, disturbance in ion homeostasis, and interference with cell migration and mobility. Furthermore, the review expounds upon the potential impact of MNPs on various organs and systems, including the brain and nervous system, heart and circulatory system, liver, spleen, lymph nodes, skin, urinary, and reproductive systems.

## 1. Introduction

Magnetic iron oxide nanoparticles (MNPs), encompassing magnetite (Fe_3_O_4_) and maghemite (γ-Fe_2_O_3_), are composed of magnetic domains with permanent magnetization. They represent a unique class of nanostructures extensively utilized in the medical field. Their ferromagnetic properties distinguish them from other nanoparticle types, including polymers, metallic elements (e.g., gold or silver), dendrimers, or carbon tubes. MNPs can be precisely directed to specific locations within the body through the influence of an external magnetic field, rendering them valuable as drug delivery systems, particularly for targeted delivery [1,2,3,4]. MNPs have well-established applications, notably in magnetic resonance imaging (MRI) and hyperthermia [5,6]. They are also employed in gene therapy [7], radiolabelling, and internal radiotherapy [8]. Specific iron oxide nanoparticle variants address microbial infections [9,10]. Recent research investigations have increasingly explored the potential of MNPs as valuable tools within the domain of bone tissue engineering [11].

The noteworthy attribute of magnetic nanoparticles for medical applications lies in their capacity for desired activity and biocompatibility within the organism. Biocompatibility is defined as the absence of toxicity, injurious effects, or physiological reactivity about living tissue or systems and the absence of immunological rejection. Well-engineered magnetite and maghemite nanoparticles are considered safe nanostructures, as iron is a naturally occurring trace element in the body. Upon digestion within lysosomes, the released iron ions can integrate into the natural circulation of this element within the organism. It is posited that the biodegradation mechanism of magnetic nanoparticles bears resemblance to the metabolism of ferritin, a cellular iron storage protein [12,13]. Consequently, several clinically approved iron oxide magnetic nanoparticles are employed in humans as magnetic imaging agents (e.g., GastroMark^®^ and Feridex^®^), magnetic hyperthermia agents (NanoTherm^®^), or therapeutic agents for the treatment of iron deficiency (Feraheme^®^). Nonetheless, the potential toxicity or lack thereof of magnetic nanoparticles is influenced by various factors, including their size, “shell” type, charge, dose, exposure time, and the type of cells or tissues exposed to the nanoparticles. Therefore, comprehensive toxicity studies for each specific type of designed nanoparticles are imperative.

The impact of iron oxide nanoparticles on oxidative stress and their correlation with cell apoptosis is widely acknowledged [14]. The surface coating of the core of magnetic nanoparticles plays a crucial role in mitigating this process [15,16,17,18]. Therefore, proper surface functionalization of MNPs not only enhances nanoparticle stability and offers functional groups for the attachment of specific drugs and desired biomolecules but also reduces or eliminates their toxicity within the body [19,20,21].

This review provides a comprehensive and detailed overview of the most current understanding of the potential toxicity mechanisms of magnetic nanoparticles at the cellular level and their impact on individual organs and systems. The analysis is designed to be accessible to readers of varying expertise while maintaining high scholarly integrity.

## 2. The Primary Mechanisms of Toxicity of MNPs at the Cellular Level

### 2.1. Oxidative Stress

**Figure 1:**
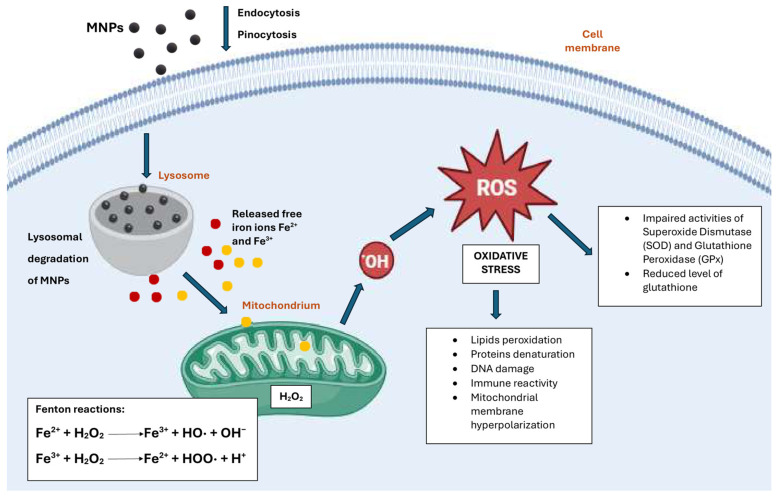
Mechanisms of MNP-mediated oxidative stress. Created with BioRender.com.

The internalization of magnetic nanoparticles by cells occurs through phagocytosis or pinocytosis after opsonization, facilitated by specific proteins [22,23,24,25]. Opsonization renders MNPs visible to specific cells, allowing them to attach to the cell surface via receptor–ligand interactions. The resulting phagosome or endosome is internalized within the cytoplasm, where it fuses with lysosomes containing enzymes in an acidic environment. Consequently, iron ions are released and can permeate nuclear or mitochondrial membranes, participating in the Haber–Weiss, Fenton, and Fenton-like reactions, which are recognized as primary mechanisms for generating reactive oxygen species (ROS) such as superoxide anions, hydroxyl radicals, and hydrogen peroxide (Figure 1) [26]. ROS, in turn, can disrupt membrane structure, impair mitochondrial and other organelle functions, and cause genetic material damage, ultimately initiating cell death pathways [27,28,29]. Pongrac et al. investigated the levels of intracellular glutathione, mitochondrial membrane potential, cell membrane potential, DNA damage, and the activities of superoxide dismutase (SOD) and glutathione peroxidase (GPx) in murine neural stem cells (NSCs) after exposure to MNPs [30]. It was indicated that the cells had reduced levels of glutathione and impaired activities of SOD and GPx. Additionally, the mitochondria experienced membrane hyperpolarization, disfunction of cell membrane potential, and DNA damage.

Multiple research studies have investigated the generation of reactive radicals ·OH by MNPs. One study by L. Wu et al. reported that MNPs with a diameter below 5 nm exhibited high toxicity towards several organs, particularly the heart, at a dosage of 100 mg/kg [31]. In contrast, nanoparticles with a size above 5 nm did not demonstrate apparent toxicity. In turn, H. Ying et al. found that 30 nm MNPs induced greater free radical production in mouse primary macrophages compared to 10 nm nanoparticles, contradicting the findings of Wu et al. [32]. Notably, various cell lines and organs exhibit distinct responses to exposure to iron oxide nanoparticles, indicating different susceptibilities to oxidative stress. Increased generation of ROS due to MNP exposure has been observed in various cell types, including MCF-7 cells [31], mice primary macrophages [32], rat lymphocytes [33], human brain-delivered endothelial cells [34], Chinese hamster lung cells [35], osteosarcoma cells [36], brain microglia cells [37], Chinese hamster ovary CHO-K1 cells [38], and human lung A549 cells [39]. Conversely, studies by Hohnholt [40], Lindemann et al. [41], and Remya et al. [42] reported no significant ROS formation after treatment with MNPs.

Applying effective antioxidants is a significant approach to suppressing the generation of ROS. For instance, a recent study highlighted the protective role of oleic acid in mitigating oxidative stress in endothelial cells induced by silica-coated MNPs approximately 19 nm in size [43]. Furthermore, the mitigating effect of ascorbic acid was demonstrated in human hepatocellular carcinoma HepG2 and human lung adenocarcinoma cells [44]. Recent advancements have led to the development of inorganic nanoparticles with antioxidant properties, known as nano-antioxidants [45]. These properties are demonstrated by metal nanoparticles such as silver and gold and transition metal oxides, including copper oxide, nickel oxide, and magnetic iron oxide. The nanoparticles are modified with antioxidants or antioxidant enzymes such as superoxide dismutase, catalase, or oxidase.

The antioxidant properties of nanoparticles are influenced by various factors, including their synthesis method, chemical composition, stability, surface area, size, coating, and charge [46]. Further modification with various phytochemicals has been observed to augment the antioxidant activity of MNPs. Despite the typical generation of free radicals by nanoparticles, their green synthesis enables them to exhibit antioxidant activity [47]. For example, research has shown that magnetic iron oxide nanoparticles synthesized using *Blumea eriantha* demonstrated an antioxidant activity level of 74.94% [48]. Nevertheless, the mechanisms underlying the antioxidant activity of nanoparticles are not yet fully comprehended and are currently the subject of intensive research [47].

The utilization of magnetic nanoparticles to induce ROS holds significant promise as a prospective approach in cancer therapy [14]. Elevated levels of free radicals within cells can propagate deleterious effects on cellular components, leading to cellular demise [49,50]. The targeted strategy of cancer treatment necessitates the selective impact of MNPs on cancer cells while preserving the integrity of healthy cells. This targeted effect can be achieved by leveraging the varying ROS generation capacity of MNPs across different cell types. Emerging evidence has underscored the discriminatory action of MNPs in regulating ROS levels in tumor and normal cells. Notably, Ahamed et al. demonstrated the selective induction of apoptosis in cancer cells through the p53 pathway by MNPs, with no discernible toxicity observed in normal cells. Cytotoxicity of Fe_3_O_4_ MNPs was examined against two types of cancer cells (human hepatocellular carcinoma HepG2 and human lung adenocarcinoma A549) and two normal cells (human lung fibroblast IMR-90 and rat hepatocytes) [44]. Similarly, Jahanbani et al. reported that MNPs stimulated ROS production and mitigated succinate dehydrogenase activity in complex II of mitochondria isolated from cancerous oral tongue squamous cells (OTSCC rat model and normal rat), while exerting negligible effects on control mitochondria [51]. Furthermore, Shi et al. innovatively developed NanoTrail, wherein the immobilization of Apo2 ligand or tumor necrosis factor (TNF)-related apoptosis-inducing ligand (Apo2L/TRAIL) on MNPs was undertaken to obviate p53-dependent apoptosis within resistant SW-480 and sensitive HCT-116 cells [52]. Exploration of this approach revealed an upregulation of the death receptor DR5 by ROS induction via MNPs, leading to amplified TRAIL/Apo2L-based apoptosis and consequential tumor cell death, with minimal adverse impact on healthy organs. In essence, while the instigation of oxidative stress by magnetic nanoparticles may bear unfavorable consequences if it compromises the viability of healthy cells, meticulous design refinements hold the potential to attenuate their cytotoxicity. Conversely, MNPs exhibit the capacity for targeted modulation, selectively obliterating cancerous cells while preserving healthy cells. This unique property bears substantial promise in the advancement of anti-cancer therapeutic modalities.

The mitochondria are the primary site of ROS production and are particularly susceptible to oxidative stress [53]. Through the induction of oxidative stress, magnetic nanoparticles can result in the impairment and dysfunction of these organelles, leading to alterations in the cell membrane’s polarity and the subsequent release of cytochrome C, culminating in apoptosis [54]. Mitochondrial depolarization permits the influx of calcium ions, which subsequently activate the modification and opening of mega-channels (MPTP) located at the inner and outer mitochondrial membrane junction. Comprising voltage-dependent ion channels (VDAC), adenine nucleotide translocase (ANT), and cyclophilin D (CyD), MPTPs undergo modification due to ROS affecting ANT thiol groups. Moreover, pro-apoptotic proteins, Bax and Bak, interact with VDAC, leading to an increase in pore size and the facilitation of cytochrome c release, thereby triggering the caspase 3-dependent apoptosis pathway [55]. Furthermore, free radicals not only impact the oxidation of mitochondrial membranes but also elevate the levels of toxic lipid peroxidation products, including malondialdehyde (MDA) and 4-hydroxy-2-nonenal [56]. Additionally, the presence of ROS leads to mitochondrial DNA damage, disrupting the function of critical mitochondrial enzymes, such as SOD1, SOD2, pyruvate dehydrogenase, aconitase, and L-ketoglutarate dehydrogenase [57]. Elevated levels of free radicals and mtDNA oxidation have been identified in patients with Alzheimer’s disease [58]. Moreover, it has been observed that ROS bolster the activity of secretase enzymes involved in amyloid production, an aberrant protein associated with Alzheimer’s disease. The excessive presence of this protein exacerbates neuronal dysfunction, neurodegeneration, and cognitive impairment in individuals with Alzheimer’s disease [59,60,61]. Additionally, oxidative stress in the mitochondria damages subunits of the mtDNA electron transport chain (ETC), ultimately leading to reduced ATP production.

### 2.2. Genotoxic Effects

The mechanisms of genotoxicity can be classified into two groups: primary genotoxicity (direct or indirect) and secondary genotoxicity, which is associated with inflammatory cells such as macrophages and polymorphonuclear leukocytes [62]. The genotoxic effect of magnetic nanoparticles is primarily attributed to their direct interaction with the DNA in the nucleus (nDNA), leading to disruptions in genetic material functioning, including interruptions in nDNA chains, nucleotide oxidation, and perturbations in transcription and replication [63,64]. A study by Shahabadi et al. examined the binding of Fe_3_O_4_@SiO_2_-nicotinamide nanoparticles to calf-thymus DNA (ct-DNA), demonstrating non-covalent interactions such as electrostatic binding, hydrogen bonding, and channel surface binding [65]. This suggests the potential applicability of these nanoparticles in cancer treatment. Another study illustrates that MNPs that are functionalized with glucose and conjugated with Coumarin (Fe_3_O_4_@Glu-Coumarin MNPs) stimulate the expression of *CASP-8*, *p53*, and *MAPK-1* genes while inhibiting the *CASP-9* and *mTOR-1* genes in liver cancer cells [66]. It is important to note that caspases coded by *CASP* comprise a group of cysteine proteases central to apoptotic responses [67]. The initiation of apoptosis is triggered by the activation of Caspase 8. Hence, the overexpression of the *CASP-8* gene led to apoptosis. The *p53* gene acts as a tumor suppressor in human cells, playing a crucial role in cell cycle regulation and initiating cell apoptosis [68]. Consequently, the elevated expression of the *p53* gene in liver cancer cells due to magnetic nanoparticles resulted in anti-proliferative effects. Conversely, *mTOR-1* primarily regulates cell growth and metabolism. Therefore, Shokrollahi et al. have concluded that Fe_3_O_4_@Glu-Coumarin MNPs can effectively inhibit the activation of mTOR-1 signaling pathways, thereby impeding the proliferation and progression of the liver HepG2 cancer cells [66]. Additionally, Siddiqui et al. have observed significant DNA damage in human umbilical vein endothelial cells (HUVECs) caused by MNPs [69]. Furthermore, exposure to MNPs at concentrations of 100 µg/mL led to the upregulation of proapoptotic genes such as *p53*, *bax*, and *CASP-3* and the downregulation of the antiapoptotic gene *bcl-2* in HUVECs.

Conversely, MNPs can indirectly induce genotoxicity through oxidative stress and excessive production of ROS, resulting in DNA damage. However, in most cases, oxidative stress is found to have a limited impact on the induction of genotoxicity by surface-modified MNPs [70]. In the secondary mechanism, activation of phagocytes in the immune response leads to increased oxygen consumption, consequentially releasing H_2_O_2_ due to activation of the NADPH-oxidase system [71].

### 2.3. Cytoskeleton Disruption

The integrity of the cytoskeleton is vital for cell viability, and any impairment to its structure can lead to cell demise. Actin filaments are primarily responsible for upholding cell architecture and facilitating organelle and vesicle transport. Disruption of these filaments can instigate cell cycle arrest and apoptosis [72]. Instances of cytoskeletal impairment due to oxidative stress have been reported [54], with evidence of non-reactive oxygen species mechanisms influencing the action of magnetic nanoparticles on microtubule proteins [73]. Acknowledging the varied cellular sensitivity to MNPs’ impact on the cytoskeleton is imperative, analogous to their distinct responses to oxidative stress. This sensitivity diversity holds potential significance in anticancer therapy. For instance, an investigation led by Master et al. examined the effects of 7–8 nm MNP–polymer complexes on the cytoskeleton of multiple cell lines, including MDA-MB-231 (human triple-negative mammary gland adenocarcinoma), BT474 (human breast ductal carcinoma), and MCF10A (human non-tumorigenic mammary gland cells) [74]. Cells containing internalized magnetic nanoparticles underwent exposure to alternating current (ACur) magnetic fields, inducing mechanical disruption of lysosomes, consequently leading to lysosomal membrane permeabilization and subsequent cell death [75,76]. Notably, the investigation revealed that the BT474 cells exhibited increased sensitivity to the treatment compared to the MDA-MB-231 cells, while the healthy MCF10A cells displayed no discernible effect [74]. In contrast, research conducted by Královec et al. has demonstrated that thiol-functionalized silica-coated magnetic nanoparticles disrupt the actin and microtubule cytoskeleton networks in healthy human lung epithelial cells [77].

### 2.4. Cell Membrane Disruption

All cells are characterized by a membrane electric potential resulting from an ion gradient and selective permeability [78]. Under standard conditions, the ion gradient typically establishes a potential ranging from −10 to −100 mV with a net negative charge on the cytosolic side. Cells possessing a physiological membrane potential are referred to as polarized, while membrane depolarization occurs when the potential is diminished or eliminated. Multiple studies have documented cell membrane depolarization induced by magnetic nanoparticles, leading to alterations in ion channel activity and disruption of cell membrane integrity. For instance, Pongrac et al. observed dose-dependent depolarization of mouse stem cells shortly after exposure to poly(L-lysine)-coated maghemite nanoparticles [79]. In another investigation, two-electrode voltage clamp experiments demonstrated that starch-coated MNPs reduced currents of the Kv1.3 and Kv7.1 potassium channels in human monocytes [80]. Importantly, these direct depolarization effects are specific to particular cell types (Jurkat T and Human Embryonic Kidney 293 cells) [81,82].

### 2.5. Changes in Cell Cycle

The cellular division process begins with the mitosis (M) phase, comprising the division of cytoplasm and DNA, forming two distinct cells. This is succeeded by the G1 phase, characterized by cellular expansion, organelle duplication, and RNA, protein, and enzymes synthesis. Subsequently, the S phase involves complete replication of the cell’s DNA, and the G2 phase is marked by the cell’s preparatory activities for mitosis, including the production of specialized proteins and RNA. Following the G1 phase, a cell may enter the G0 phase, a resting phase, rather than advancing to the DNA synthesis stage (S). Studies suggest that magnetic nanoparticles have the potential to induce alterations in the cell cycle. For instance, Shokrollahi et al. observed that exposure to Fe_3_O_4_@Glu-Coumarin MNPs prevented liver cancer HepG2 cells from progressing through the G1 checkpoint, resulting in cell death by apoptosis [66]. MNPs were also found to cause cell cycle arrest in human umbilical vein endothelial cells (HUVECs) [69]. Furthermore, MNPs loaded with doxorubicin (DOX) were shown to restrict the G2 phase in breast cancer (MCF7) cells [83]. Importantly, bare MNPs did not induce any alteration in the G2 phase compared to the control. Tamoxifen-loaded L-lysine-coated MNPs induced cell cycle arrest at the G0/G1 phase in MCF7 cells [84]. Moreover, Majeed et al. reported that engineered green iron oxide nanoparticles with l-arginine exhibited significant toxicity towards breast cancer (MDA-MB-231) cells, leading to cell cycle arrest at the G2/M phase and consequential DNA damage while causing minimal harm to normal cells [85].

### 2.6. Inflammatory Response

When considering the application of magnetic nanoparticles for medical purposes, it is crucial to evaluate their potential influence on essential innate immune responses. Numerous studies have delved into this subject. For instance, Grosse et al. examined the effects of MNPs coated with organic layers comprising a monolayer of oleic acid and a monolayer of amphiphilic polymer on primary human monocytes in the presence and absence of the Toll-like receptor 4 (TLR4) agonist lipopolysaccharide (LPS) [86]. TLR4 is a receptor that serves as a sensor for lipopolysaccharide (LPS), and its activation leads to the production of several pro-inflammatory, antiviral, and anti-bacterial cytokines [87]. The researchers observed that the tested MNPs did not influence the production of proinflammatory cytokines such as tumor necrosis factor-α, interleukin-6, and interleukin-1β. However, they noted suppression of LPS-induced nuclear factor kappa B activation and the production of proinflammatory cytokines in a dose-dependent manner. In another study, the thromboinflammatory response of uncoated MNPs with a size of 10–30 nm was investigated in whole blood in interaction with human endothelial cells [88]. The MNPs were found to elicit a potent thromboinflammatory response, as evidenced by a significantly increased release of 21 out of 27 analyzed cytokines. Furthermore, MNPs significantly increased the activation markers of endothelial cells, P/E-selectin, monocytes, granulocytes, and platelets. The experiments also demonstrated cytotoxic effects, as evidenced by elevated levels of lactate dehydrogenase (LDH) and heme. In a related study, Chauhan et al. investigated the potential application of vitamin K_3_-loaded magnetic nanoparticle-mediated synergistic magneto-thermodynamic therapy (MTD) for cancer treatment (ectopic tumor model of A549 lung adenocarcinoma) [89]. The results indicated that the examined MNPs not only induced the generation of ROS but also led to increased expression levels of heat shock proteins and proinflammatory cytokines (IL-6, TNF-α, IL-1α, IL-1β).

### 2.7. Disturbance in Iron Homeostasis

The degradation of MNPs within lysosomes results in the release of iron ions, which subsequently become integrated into the body’s natural iron circulation. Free iron is then sequestered as ferritin, a protein that binds with Fe^3+^ ions and stores them in the liver [12,13]. Another protein, transferrin, regulates the concentration of iron ions in the blood plasma and transports them to the tissues. Exceeding the iron storage capacity of these proteins leads to iron overload, disrupting iron homeostasis. Iron overload is defined by elevated ferritin levels (>300 ng/L) and heightened transferrin saturation (>40%) [90]. The excess iron is released into the plasma and circulates in an unbound, redox-active form, causing cellular damage and culminating in organ failure in advanced stages [91,92].

### 2.8. Disturbance of Cell Migration and Mobility

Alterations in gene regulation and damage to the cytoskeleton can disrupt cell migration [93]. This phenomenon has been observed in endothelial and endothelial progenitor cells [94,95], neural stem cells [96], and macrophages [97]. According to Mohsin et al., the cellular uptake of MNPs markedly increased cell mobility and contraction due to the high levels of intracellular nanoparticles, leading to increased intracellular tension and regulation of cell behavior [98].

**Figure 2:**
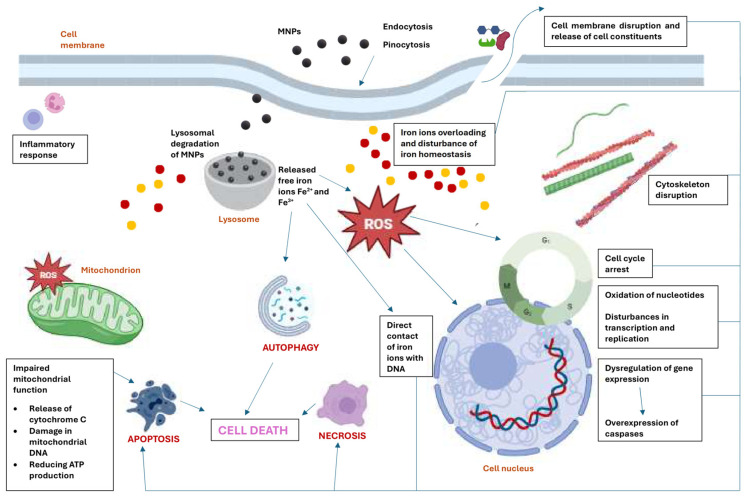
Mechanisms of MNP-mediated toxicity at the cellular level. Created with BioRender.com.

The main mechanisms of MNP toxicity at the cellular level are depicted in Figure 2.

## 3. Systemic and Organ-Toxic Effects of MNPs

### 3.1. Brain and Nervous System

The blood–brain barrier (BBB) and the blood–cerebrospinal fluid (CSF) barrier act as pivotal defense mechanisms, regulating the passage of substances from the blood into neurons. Their selective permeability restricts the entry of various compounds, allowing only essential substances such as glucose and amino acids to support the optimal functioning of the nervous system. Studies have demonstrated the potential for magnetic iron oxide nanoparticles to traverse the BBB, dependent on their physicochemical and surface properties. However, accurately predicting whether specific nanoparticles can effectively penetrate neurons remains a significant challenge [99]. In vitro studies on the penetration of the BBB by MNPs under a static magnetic field are primarily conducted by administering nanoparticles of various sizes and characteristics to in vitro BBB models created through the co-culturing of endothelial cells and astrocytes [100]. One study used brain capillary endothelial cells (BCECs) that were incubated with MNPs at various concentrations (35, 70, and 140 µg/mL) for 24 h [101]. The study findings revealed that both internalization and transcellular transport processes occurred in the absence of magnetic fields without inducing any toxic effects. Consequently, the authors concluded that the MNPs did not disrupt the barrier’s integrity, did not affect cell viability, and were successfully internalized by astrocytes. Recently, Shin and Lee conducted a detailed study on the effects of silica-coated magnetic nanoparticles containing rhodamine B isothiocyanate dye (MNPs@SiO_2_(RITC)) on BV2 murine microglial cells [102]. Additionally, the authors examined amyloid beta (Aβ) accumulation and molecular changes using integrated transcriptomics, proteomics, and metabolomics (triple-omics) analyses. Following the administration of 0.1 μg/μL MNPs@SiO_2_(RITC), a significant increase in the amount of Aβ was observed. This discovery points to a notable association between nanotoxicity and Aβ accumulation. Furthermore, the MNPs@SiO_2_(RITC)-treated BV2 cells displayed evidence of lysosomal swelling, a reduction in proteolytic activity dependent on dosage, and an elevation in lysosomal swelling- and autophagy-related protein levels. Additionally, a decrease in proteasome activity was observed in the BV2 cells following treatment with MNPs@SiO_2_(RITC), leading to a subsequent reduction in intracellular adenosine triphosphate (ATP).

In the context of neurotoxicity research, in vivo studies were undertaken to investigate the impact of MNPs, with specific experiments being conducted using Wistar rats [103]. The animals were administered intravenously by green, carob leaf-synthesized, negatively charged MNPs with a hydrodynamic diameter of approximately 80 nm. The study showed that MNPs had the most significant impact on the hippocampus and striatum of the brain, leading to moderate neuronal degeneration. Additionally, mild neuronal degeneration in the cerebral cortex and slight degeneration in the cerebral cortex were observed. In a separate study, four different types of nanoparticles were administered to Sprague-Dawley rats: dimercaptosuccinic acid (DMSA)-coated MNPs (both γ-Fe_2_O_3_ and Fe_3_O_4_), PEG-coated Fe_3_O_4_ nanoparticles, and PEG-Au-coated Fe_3_O_4_ nanoparticles [104]. Neural MAPK/ERK and Caspase-3 levels were analyzed after intraneural injection, indicating inflammation and apoptosis. Both markers exhibited a significant increase in all animals injected with magnetic nanoparticles. However, there are also reports indicating the absence of significantly toxic effects of specific types of magnetic nanoparticles on the brain. For example, a study by J. S. Kim et al. involved the treatment of male ICR mice with silica-coated MNPs [105]. The mice were intraperitoneally administered three different concentrations of MNPs@SiO_2_, namely 100, 50, and 25 mg/kg, over 4 weeks. The study’s findings established the capability of MNPs@SiO_2_ to penetrate the blood–brain barrier (BBB) without compromising its functionality. Moreover, histological examinations and clinical biochemistry tests revealed no notable changes or macroscopic lesions in the organs, including the brain, in the MNPs@SiO_2_-treated groups.

Intriguingly, magnetic nanoparticles may alleviate the toxicity of drugs in the brain. Fouad et al. tested the neuroprotective potential of sulforaphane (SF) loaded within Fe_3_O_4_ against cisplatin-induced neurotoxicity [106]. The study demonstrated that SF-MNPs significantly reduced acetylcholinesterase activity, alleviated oxidative stress, and ameliorated behavioral outcomes. These results were corroborated by histopathological features, confirming the safe toxicological profile of Fe_3_O_4_ nanoparticles.

The research findings on the neurotoxicity of magnetic iron oxide nanoparticles are often contradictory. This inconsistency can be attributed to a range of factors that influence the potential toxicity of the nanoparticles, including their physicochemical properties, surface chemistry, size, dosage, method of administration, and experimental model. Notably, several studies have indicated the potential neurotoxicity of MNPs, underscoring the necessity for further comprehensive investigations to elucidate and characterize this impact.

### 3.2. Heart and Circulatory System

The specific molecular mechanisms of cardiotoxicity of magnetic iron oxide nanoparticles are not yet fully understood. However, it is widely accepted that the primary cause of heart toxicity is the generation of free radicals by these nanoparticles. In vitro experiments have revealed that nanoparticles significantly elevate oxidative stress damage, leading to overactivated autophagy and endoplasmic reticulum stress, ultimately resulting in cardiomyocyte apoptosis [107]. A proposed hypothesis suggests that the inclusion of antioxidants could effectively mitigate the cardiotoxic effects of nanoparticles. N-acetylcysteine (NAC), known for its potent antioxidant properties, was integrated into mesoporous silica nanoparticles (M-MSN) to create M-MSN@NAC with a magnetic (Fe_3_O_4_) core [107,108,109]. The M-MSN@NAC treatment significantly reduced the oxidative stress of H/R cardiomyocytes induced by MNPs. This effect was attributed to the release of NAC, which effectively restricted the formation of peroxidation products. Significantly, the negatively charged nanoparticles did not substantially impact the actin skeleton of the heart cells, in contrast to their detrimental effect on the brain [110]. Nevertheless, adverse effects and cardiotoxicity associated with iron oxide nanoparticles have also been documented. For example, research has shown that the intravenous administration of polyacrylic acid-coated γ-Fe_2_O_3_ nanoparticles resulted in a decrease in mean arterial blood pressure (MAP) in healthy BALB/cJ mice [111]. In pigs, dimercaptosuccinic acid-coated MNPs (12 nm) administered intravenously at doses of 0.5 or 2.0 mg/kg induced a transient but significant reduction in MAP [112]. In a separate study, Manickam et al. tested the cardiotoxicity of Fe_2_O_3_-MNPs (<50 nm) in mice [113]. A significant accumulation of MNPs was detected in the hearts of animals after 30 days of administration at doses of 25 and 50 mg/kg. Exposure to these particles resulted in oxidative myocardial damage. As a result, the researchers observed harmed mitochondria, decreased ATP levels, and overexpression of NOX4. Additionally, the authors suggested that mice treated with 50 mg/kg experienced both necrosis and apoptosis. Nemmar et al. examined the impact of 5 nm Fe_3_O_4_ with PEG stabilizing ligands on the heart and circulatory system [114]. Following the intravenous administration of nanoparticles at doses of 2 µg/kg and 10 µg/kg, a notable reduction in thrombotic occlusion time was observed in BALB/C mice’s pial arterioles and venules. Furthermore, the presence of Fe_3_O_4_ nanoparticles led to a marked and dose-dependent increase in plasma levels of LDH, creatine phosphokinase-MB (CK-MB), and troponin-I, indicative of myocardial membrane damage. Elevated levels of ROS and DNA damage in the heart tissue were also noted at all studied doses of the nanoparticles.

Several reports have suggested that the immobilization of anticancer drugs on magnetic nanoparticles (MNPs) or the construction of carrier systems with a magnetic core may lead to a reduction in the cardiotoxicity of cytostatics. For example, Shetake et al. developed nano-formulations of magnetic nanoparticles co-encapsulated with doxorubicin (DOX) and indocyanine green (ICG) in a liposomal carrier (T-LMD), demonstrating minimal toxicity to heart tissue [115]. This is in contrast to DOX, which is known for its significant cardiotoxic effects [116,117]. In another study, a curcumin-loaded magnetic hydrogel nanocomposite was found to exhibit cardioprotective effects against doxorubicin-induced cardiac toxicity in rat cardiomyocytes [118]. Additionally, MNPs coated with DOX-conjugated heparin were found to be significantly less harmful to cardiac tissue in mice compared to free DOX at the same dosage [119]. Moreover, Xiong et al. designed and evaluated 2,3-dimercaptosuccinic acid-coated MNPs as potential agents for the treatment of cardiovascular diseases, concluding that these nanostructures showed promise as nanomaterials for protecting the heart from ischemic damage [120].

### 3.3. Liver, Spleen, and Lymph Nodes

The comprehensive investigation of magnetic nanoparticles’ toxicity towards the liver and spleen is essential, as these organs serve as the primary capture and biodistribution sites for MNPs in both the short and long term [121]. Kupffer cells, a specialized population of liver macrophages, are responsible for the phagocytosis of pathogens, including MNPs, entering from the bloodstream. Additionally, the spleen plays a crucial role in removing pathogens and foreign particles, making it important to understand the impact of MNPs on this organ. Moreover, when administered intramuscularly or subcutaneously, regional lymph nodes may serve as the initial filter site for MNPs. High levels of alkaline phosphatase (ALP), alanine aminotransferase (ALT), and aspartate aminotransferase (AST) are typically indicative of liver damage. Additional markers for hepatic impairment involve hepatic plasma malondialdehyde (MDA), lactate dehydrogenase (LDH), tacrolimus (TAC), glutathione reductase (GSH), and gamma-glutamyl transferase (GGT) [122]. Examination of these enzyme profiles is typically performed in toxicity studies. Furthermore, histological analysis of the liver is considered a standard procedure. Askri et al. conducted a study investigating the impact of exposure to sub-acute MNPs (30 nm) at a 10 mg/kg dosage on Wistar rats [123]. The study revealed a notable decrease in alkaline phosphatase levels in rats exposed to MNPs, while no significant changes were observed in the levels of other hepatic enzymes (ALT and AST). In another study, the hepatotoxicity of dextran-coated iron oxide nanoparticles and dextran-coated MNPs conjugated with quercetin was studied in Wistar rats [124]. The study results revealed that there were no significant differences in the levels of AST, ALT, ALP, GGT, and LDH between the control group and the groups that received 50 and 100 mg/kg of quercetin-conjugated, dextran-coated MNPs in the liver. Administering dextran-coated magnetic nanoparticles at 100 mg/kg markedly reduced hepatic GSH level and CAT activity. Furthermore, there was a significant increase in hepatic MDA levels, while the hepatic TAC, GSH levels, MDA levels, and CAT activity exhibited no significant variances compared to the control group. These findings support the conclusion that quercetin effectively mitigated the toxic effects of MNPs and safeguarded the liver from oxidative damage. In a separate study, an investigation was conducted on the toxicity of 10 nm MNPs coated with the fourth generation (G4) of polyamidoamine (PAMAM) dendrimer toward BALB/c mice [125]. The histopathological results revealed edema and cytoplasmic depletion in the liver cells at a dosage of 10 mg/kg. Yaremenko et al. tested the toxicity of magnetic nanoparticles (50 nm) coated with glucuronic acid in BALB/c mice [126]. The histological analysis indicated that the injection of nanoparticles had no discernible effect on liver morphology on days 1, 7, 28, and 56. However, on day 14 post-injection, an increase in non-epithelial cells, particularly Kupfer cells and lymphocytes, was observed. Additionally, hemosiderin deposits were noted on days 1, 7, and 14 following the injection. Subsequently, the number of iron-positive cells decreased after 28 days, and on day 56, only small extracellular deposits of hemosiderin were evident, likely attributed to the demises of macrophages loaded with the nanoparticles. In the spleen, no morphological pathologies were observed on day 1 post-nanoparticle injection. However, after 7 days, hyperplasia of the white pulp and an increase in lymphoid nodules were noted. After 56 days, an increase in red pulp cellularity and the number of megakaryocytes was observed. Similar to the liver tissue, hemosiderosis of the spleen was observed throughout the 8-week experiment post-Perls staining. Paulini et al. investigated the toxicity of DMSA-coated magnetic nanoparticles (11 nm) in rats, administered at doses of 0.5 or 5 mg Fe/kg, with long-term monitoring [127]. It was reported that no changes in serum ALT and AST were associated with DMSA-MNP administration. Furthermore, all animals showed normal liver and spleen histology 10 months after DMSA-MNP injection. However, recent studies have indicated that the administration of Fe_3_O_4_ nanoparticles, synthesized using *D. mucronate* leaves, at 100 or 300 mg per kg of a mouse’s body, induced alterations in the liver and spleen tissues, with more pronounced effects observed at the higher dosage [128]. In these tissues, edematous, dilation of blood vessels and sinusoids were observed. Additionally, sinusoids have lost their original organization due to bio-Fe_3_O_4_ MNP (300 mg/kg) accumulation compared with control and 100 mg/kg bio-Fe_3_O_4_ MNP injection.

Magnetic nanoparticles exhibit substantial accumulation in lymph nodes, thus holding promise for early cancer detection in the lymphatic tissues. MNPs with a hydrodynamic diameter ranging from 20 to 30 nm demonstrate an extended circulation period, rendering them valuable for applications in lymphography, inflammation, and blood pool imaging [129]. Extensive research is currently underway to explore the potential application of MNPs as nanotheranostics in the treatment of cancer lymph node metastasis [130]. Regrettably, there is a paucity of comprehensive studies on the potential toxicity of MNPs to lymph nodes, and the available studies on this matter are notably limited. A particular report evaluated carboxylic mannan-coated MNPs (CM-MNPs) with a 34 nm size, designed to target immune cells for lymph node-specific MRI in vivo [131]. The mice administered with CM-MNPs did not show any abnormalities. The LD50 of CM-MNPs was found to be more than 80 mg Fe/kg in mice, indicating the safety of the studied nanostructures. Sekino et al. developed a handheld magnetic probe with a permanent magnet and Hall sensor to identify sentinel lymph nodes (SLNs) in breast cancer patients [132]. Histopathology confirmed the accumulation of the MNPs (Resovist, 60 nm, carboxydextran shell) in the cortex of the extracted node. However, the authors did not present possible changes or potential toxicity effects caused by nanostructures in this tissue. In another study, polyacrylic acid-modified iron oxide nanoparticles (PAA@MNPs) were studied as potential agents for differentiating between hyperplastic and metastatic breast cancer lymph nodes [133]. The authors reported no negative effects on body weight, hematology, coagulation parameters, serum biochemistry, and gross anatomy of all Sprague-Dawley rats after receiving single or multiple doses of PAA@MNPs via intravenous administration. Additionally, histopathological examination revealed no abnormal findings.

### 3.4. Respiratory System

Magnetic iron oxide nanoparticles are the subject of extensive research due to their potential applications in lung disease diagnostics and therapies. Therefore, it is crucial to thoroughly examine their potential toxicity to lung tissue. Kubovcikova et al. examined the potential of N-acetylcysteine (NAC) immobilized on poly-L-lysine-functionalized iron oxide magnetic nanoparticles (NAC-PLL-MNPs, 10–11 nm) for enabling magnetic resonance imaging of drug distribution in the lungs [134]. NAC is a pharmaceutical approved by the FDA and recognized by the WHO as an essential medication. It is extensively utilized for treating acetaminophen overdose and, more recently, as a mucolytic agent in respiratory diseases [135]. PLL-MNPs and NAC-adsorbed PLL-MNPs displayed excellent MRI contrast properties, allowing for the differentiation between magnetic nanoparticles with and without immobilized NAC [134]. Cytotoxicity experiments on the obtained nanostructures were carried out using an LDH assay kit on the HEK cell line at concentrations of 0.1, 10, and 50 µg/mL. No cell toxicity was observed after 24 or 48 h of incubation at any of the three concentrations. However, it is notable that the researchers did not examine toxicity specifically in lung tissue. In a distinct study, the biological impact of green-synthesized MNPs on two different lung tumorigenic monolayers (A549 cells—lung carcinoma with an epithelial-like morphology and NCI-H460 cell line—large lung cancer cells) and a 3D normal bronchial model was investigated [136]. The MTT assay showed that 24 h after treatment, none of the test samples significantly reduced the viability of the 3D microtissues, with viability rates above 80%. The 3D models treated with MNPs exhibited some superficial layer damage, but no damage to the microporous membrane or loose epithelial junctions was observed. Additionally, no histopathological changes were noted. In conclusion, MNPs synthesized using green methods were found to be non-toxic to bronchial epithelia when applied at a concentration of 500 µg/mL. As for the antitumoral activity of the obtained MNPs on two human lung carcinoma cell lines, the Alamar blue test showed that the viability of the A549 cell population was more affected compared to the viability of NCI-H460 cells. The efficacy of tumor-targeted magnetic nanoparticles for magnetic hyperthermia treatment of non-small cell lung cancer (NSCLC) in mice via inhalation was evaluated by Sadhuka et al. [137]. The study findings demonstrated the high accumulation of EGFR-targeted MNPs (EGFR—Epidermal Growth Factor Receptor) in the pulmonary tissues. Furthermore, the administered mice exhibited no distress symptoms over the 30-day study period, suggesting the absence of acute systemic toxicity or harm to healthy lung tissue. In a separate report, the study presented results regarding the efficacy of MNPs in the treatment of bacterial pneumonia [138]. Vancomycin (VAN) was affixed to the core of magnetic nanoparticles, followed by spray-drying onto lactose/dextran. Subsequent in vivo safety and pharmacokinetic assessments validated the pulmonary tissue localization of VAN-coated MNPs and evidenced a reduction in adverse effects in comparison to free VAN.

Numerous types of magnetic nanoparticles tend to accumulate in the lungs, even when the lung tissue is not the primary target for MNPs in potential therapies for the respiratory system. For instance, several reports have documented the biodistribution of dimercaptosuccinic acid (DMSA)-coated MNPs. Typically, these nanostructures do not adversely affect cell viability in vitro [139]. One significant concern pertains to the potential impact of such nanoparticles on organs in the context of biological procedures. For instance, investigations conducted on mice demonstrated that, following intravenous injection, DMSA-MNPs were primarily concentrated in the lungs. These nanoparticles were observed in the blood vessels within the lungs, subsequently in the capillaries, and ultimately within parenchyma cells. Additionally, some nanoparticles were detected in the cytoplasm of macrophages within the bronchiolar lumen. The presence of DMSA-MNPs in the lungs contributed to heightened levels of interleukin-1 and interleukin-6, triggering an inflammatory process [140]. Mild inflammation of the lung parenchyma was observed up to 15 days after DMSA-MNP administration, with an apparent increase in the number of inflammatory cells distributed throughout the parenchyma. In a separate study, an investigation was conducted to examine the long-term impacts of DMSA-MNPs on the organs of rats [127]. The study findings indicated a mild interalveolar septal thickening in the lungs; however, the animals did not display any clinical respiratory symptoms. Askri et al. investigated the effects of sub-acute exposure to 30 nm MNPs on Wistar rats [123]. The animals were administered intranasally at a dosage of 10 mg/kg body weight. The findings indicated that MNPs had no observable effect on the structure of the lungs.

### 3.5. Urinary System

Investigating renal clearance of magnetic iron oxide nanoparticles is of significant interest in minimizing the systemic toxicity of MNPs [141]. The nanoparticles need to overcome the glomerular filtration barrier in order to be effectively eliminated by the kidneys in their pristine state. This process is particularly efficient for MNPs with a hydrodynamic diameter below 6 nm [142,143]. The research conducted by Wei et al. involved the fabrication of 3 nm Fe_3_O_4_ nanoparticles, which were subsequently coated with dopamine sulfonic acid, resulting in the formation of 4.7 nm nanostructures [144]. The obtained MNPs demonstrated good T_1_ contrast agent properties and facilitated rapid metabolism through the mice’s kidneys. Liu et al. synthesized amphoteric conjugated hollow Fe_3_O_4_ nanoparticles measuring 7 nm in size [145]. These nanoparticles held potential as T_1_ contrast agents and demonstrated rapid and complete clearance by the kidneys. Both reports’ authors confirmed the biosafety of the nanostructures. In another study, a renal-clearable ultra-small ferrite nanoprobe (UMFNPs@ZDS) was proposed for highly sensitive early diagnosis of kidney damage using structural and functional MRI in vivo [146]. The nanostructures consisted of a ferrite core coated with a zwitterionic layer and had a high T_1_ relaxivity and a small hydrodynamic size (6.43 nm). In cytotoxicity studies utilizing the MTT assay against the HK-2 cell line, which is an immortalized proximal tubule epithelial cell line derived from a normal adult human kidney, it was observed that cell viability remained above 80% even when exposed to a high concentration of UMFNPs@ZDS (200 μg/mL). Moreover, subsequent histopathological evaluation following the administration of the nanoprobe to rats revealed the absence of noteworthy lesions in vital organs, including the kidneys. However, there is an ongoing concern regarding the kidney toxicity of magnetic nanoparticles with larger sizes and different surface chemistry. Several articles have documented the potential toxicity of MNPs to various organs, including the urinary system. In a study by Alalaq et al., Fe_2_O_3_ nanoparticles of varying sizes (45 to 46 nm) were orally administered to mice every 48 h for 60 days [147]. The histological analysis of different segments of the mice’s kidneys revealed no evidence of kidney damage at lower concentrations (6 and 8 µg/L in drinking water) of nanoparticles. However, 100 and 120 µg/L concentrations resulted in noticeable structural changes and congestion between the tubules. Additionally, damage to cells in the proximal tubules and the epithelial lining of the distal convoluted tubules was observed. Another study investigated the nephrotoxicity of dextran-coated MNPs and quercetin-conjugated MNPs (QMNPs) in Wistar rats [124]. Quercetin is considered a versatile molecule due to its wide range of clinical effects, including inhibiting carcinogenesis and reducing cardiovascular diseases [148]. The study results by Kazemipour et al. showed that renal catalase (CAT) activity significantly increased in the group that received 100 mg/kg of MNP-quercetin, while it significantly decreased in the group that received 100 mg/kg of dextran-coated MNPs compared to the control group [124]. However, the levels of renal tacrolimus (TAC), glutathione reductase (GSH), and plasma malondialdehyde (MDA) were not significantly different among the groups. The 100 mg/kg dextran-coated MNPs did not cause any oxidative injury to the kidneys. In a separate study, researchers assessed the effects of 40 nm MNPs on renal function [126]. The histopathology analysis indicated no morphological changes in the organs following 28 days of administering nanoparticles to BALB/c mice. However, on day 56, a dystrophic change was observed in the epithelium of several tubules. Furthermore, after Perls staining on day one post-nanoparticle injection, diffuse blue staining was observed in the epithelium of some kidney tubules, primarily located in the luminal area of the cells. Nonetheless, these changes had disappeared by days 28 and 56. It was postulated that excess iron in the kidneys may have resulted from the non-specific accumulation of the smallest nanoparticles and their subsequent degradation. In a study by Attia and Thalij, the impact of MNPs and chitosan-coated MNPs on kidney function parameters (creatinine and urea) in male albino rats induced with anemia using phenyl-hydrazine was assessed [149]. The findings revealed elevated levels of creatinine and urea in the rats induced with anemia, indicative of renal impairment. However, the administration of MNPs and chitosan-coated MNPs to the anemia-induced rats resulted in sustained normal levels of kidney parameters.

### 3.6. Reproductive System

Magnetic iron oxide nanoparticles demonstrate promise for diagnosing and treating reproductive system diseases. For instance, MNPs attached to *Arachis hypogea* lectin/*Pisum sativum* lectin (PNA/PSA lectin) have been utilized to detect defective spermatozoa during the selection process [150]. In another study, MNPs conjugated with Annexin V and lectin were utilized to isolate apoptotic and acrosome-activated sperms from their healthy counterparts in boar and camel [151,152]. It was also reported that MNPs have proven valuable tools in monitoring human mesenchymal stem cells transplanted into the penile cavernosum of rats with erectile dysfunction [153]. In conclusion, the precise engineering of magnetic nanoparticles enables the development of safe nanoscale vesicles to treat reproductive system disorders. Nonetheless, there are documented instances regarding the adverse effects of iron oxide nanoparticles on the reproductive system. In a study conducted by Sundarraj et al., the potential toxicity followed by repeated administration of Fe_2_O_3_ nanoparticles on the testes of mice was investigated [154]. The study observed the accumulation of these nanostructures in the testes following exposure. Administration of 25 and 50 mg/kg of nanoparticles resulted in elevated levels of ROS, lipid peroxidation, protein carbonyl content, glutathione peroxidase activity, and nitric oxide, while concurrently decreasing the levels of antioxidants such as superoxide dismutase, catalase, glutathione, and vitamin C. The study also noted apoptosis, along with higher concentrations of MNP exposure, leading to an increase in serum testosterone levels. In a separate study, pregnant mice were intraperitoneally injected with varying doses of MNPs coated with dimercaptosuccinic acid (DMSA) [155]. The study results indicated that doses exceeding 50 mg/kg of DMSA-coated magnetic nanoparticles could interfere with embryo development. Al-Shammari and Al-Saaidi studied the impact of magnetic nanoparticles on male rat reproduction, considering both dosage and duration of exposure [156]. The animals were administered with MNP solution orally at doses of 1 (TL group), 5 (TM group), and 10 (TH group) mg/kg/day for 28 days. The results showed a significant decrease in the relative weights of the testis, epididymis, prostate, and seminal vesicle in the TH and TM groups compared to the control group. In contrast, no significant change was observed in the TL group. Histopathological studies revealed degenerative changes and a reduced population of germinal epithelium in the TM and TH groups. Additionally, vacuolation, necrosis, hyaline degeneration of spermatogonia, decreased number of spermatocytes, and hyperplasia of Sertoli cells were observed. Furthermore, there was a significant increase in testicular luteinizing hormone receptor (LHR) gene expression levels in the TL group, while the TM and TH groups exhibited a significant decline relative to the control group. The effect on the endocrine system was confirmed by evaluating hormone concentrations in serum. The concentrations of gonadotropin-releasing hormone (GnRH), follicle-stimulating hormone (FSH), luteinizing hormone (LH), and testosterone in male rats from the TH group were the lowest, followed by male rats from the TM group, compared to male rats in the control group.

### 3.7. Skin

One of the primary pathways for the entry of nanoparticles into the body involves absorption through the skin. Consequently, the presence of iron oxide nanoparticles can influence the functions of this organ. Penetration of MNPs into healthy skin entails the generation of free radicals, oxidative stress and collagen depletion [157,158]. Following dermal exposure to nanoparticles, subsequent physiological responses may encompass keratinization, dermal atrophy, and the manifestation of skin wrinkling. However, comprehensive investigation into the cutaneous toxicity of magnetic nanoparticles remains scarce. A study conducted by Amin et al. evaluated the cytotoxic potential of 54 nm magnetic nanoparticles on both normal and malignant human skin cells by employing the MTT assay [159]. The cell lines included human dermal fibroblasts, human squamous cell carcinoma cells (A431 cells), and human epidermal keratinocytes (HaCaT cells). The research results indicated that, within the tested concentration range (10 µg/mL to 500 µg/mL), Fe_3_O_4_ nanoparticles demonstrated negligible impact on all investigated cells.

Iron oxide nanoparticles can have great potential in the treatment of skin diseases. For instance, Duval et al. evaluated the protein and gene expression of B16 melanoma mouse cells following medium magnetic hyperthermia [160]. Melanoma cells were collected and combined with MNPs and then exposed to an alternating magnetic field (AMF). The findings demonstrated a marked upregulation of the HSP70 gene, associated with enhanced heat tolerance and immunogenicity, as well as alterations in specific receptor gene pathways linked to adsorbent chemistry and toll-like receptors. Furthermore, in a separate study, a hydrogel incorporating dextran-coated iron oxide nanoparticles (DEX-MNPs) was proposed and evaluated as a potential vehicle for topical photothermal therapy (PTT) in the treatment of skin cancer [161]. Preliminary efficacy studies in the B16F10 s.c. mice models have shown that the use of DEX-MNP gel in topical PTT can significantly reduce tumor volumes. Simultaneous treatment involving a single PTT session with 100 μgFe/mL DEX-MNP gel and applying 0.5 W laser power for 10 min resulted in an 85% significant inhibition of tumor growth.

**Figure 3:**
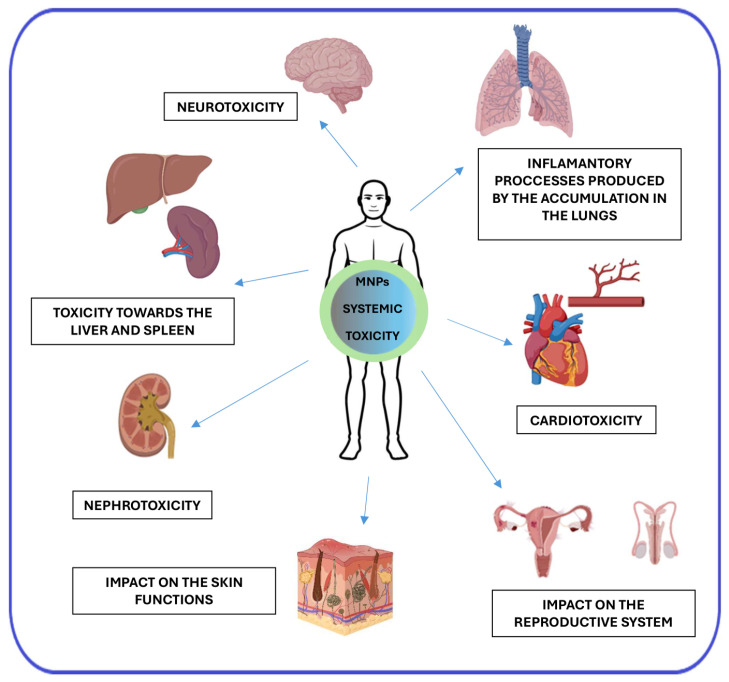
The main organs and systems affected by MNPs. Created with BioRender.com.

The main organs and systems affected by MNPs are summarized in Figure 3.

The summary of magnetic nanoparticles’ toxicity towards different models and their varied properties is presented in Table 1.

## 4. The Main Strategies for Mitigating the Toxicity of MNPs

When developing magnetic nanoparticles for medical purposes, researchers commonly adhere to fundamental principles aimed at minimizing nanoparticle toxicity. For instance, the application of surface functionalization with biocompatible polymers such as PEG is recognized for its ability to significantly diminish nanoparticle toxicity. This is predominantly rationalized by the limitation of free iron ion release and the altered interactions of MNPs with negatively charged cellular membranes [185]. In a recent study, magnetic Fe_3_O_4_ nanoparticles functionalized with (3-aminopropyl)triethoxysilane (APTES) or N-carboxymethyl chitosan (CMC) were suggested as potential nanocarriers for methotrexate (MTX) to specifically target ovarian cancer cell lines [186]. These polymer modifications were observed to effectively mitigate the toxicity of the nanoparticles, as evidenced by the lack of cell proliferation inhibition in the MTT assay.

Aptamers represent highly promising ligands for the functionalization of magnetic nanoparticles (MNPs) and play a crucial role in enhancing their biocompatibility. These are synthetic single-stranded RNA or DNA molecules, comprising 30 to 80 nucleotides, possessing the capability to specifically bind to a diverse array of molecular and cellular targets, including proteins, small organic molecules, viral particles, bacteria, antibodies, entire cells, cell lysates, and even tissues [187,188,189]. Aptamers function as functional analogs of antibodies; however, they exhibit several distinct advantages attributed to their physicochemical properties and the methodologies employed in their preparation. Among these advantages are elevated specificity, enhanced stability, reduced immunogenicity and resistance to reversible denaturation. By increasing solubility and minimizing agglomeration, aptamers significantly enhance the biocompatibility of MNPs [190].

An alternative method to modulate nanoparticle interactions with cell membranes involves the manipulation of nanoparticle morphology. Additionally, morphology may be correlated with the dissolution of the material [191]. The dissolving rate of particles is influenced by their various morphologies, surface areas, crystal planes, and particle curvature. Particles with smaller curvature radii are less energetically favorable and thus prone to undergo more dissolution. Therefore, it can be inferred that morphologies such as nanorods may exhibit lower toxicity compared to nanospheres, as their dissolution rate is expected to be slower.

One strategy for mitigating the toxicity of MNPs resulting from oxidative stress involves concurrently administering various antioxidants during nanoparticle therapy. Ucar et al. demonstrated that using ulexite (UX) at a concentration of 18.75 mg/L as a natural therapeutic agent effectively reduced oxidative stress in brain tissue [192]. On the other hand, MNPs conjugated with quercetin (QC) promoted neurogenesis without any toxicity [193]. The research findings elucidate that the QC inhibited protein aggregation and acted against iron overload via iron-chelating activity, iron homeostasis gene regulation, radical scavenging, and attenuation of the Fenton/Haber–Weiss reaction. Another study revealed that lipoic acid exhibited potent antioxidant properties by effectively scavenging hydroxyl radicals generated in the Fenton reaction involving Fe(II) [194].

## 5. MNPs in Clinical Trials

The superparamagnetic characteristics of clinically tested and approved nanoparticles are attributed to their iron oxide cores, which are predominantly composed of magnetite (Fe_3_O_4_) or maghemite (γ-Fe_2_O_3_). These cores typically range in size from 10 to 50 nanometers, which allows them to exhibit superparamagnetic behavior effectively.

A range of magnetic nanoparticles with varying characteristics have been demonstrated to be non-toxic, as evidenced by their approval for clinical applications. These nanoparticles are primarily utilized as agents in hyperthermia treatments and as contrast-enhancing agents in magnetic resonance imaging (MRI) [195,196].

Magnetic fluid hyperthermia (MFH) has undergone clinical evaluation. A number of studies have established the safety and feasibility of this technique. The administration of nanoparticles can be conducted through various methodologies, including stereotactic guidance, computed tomography (CT) guidance, ultrasound guidance, and intraoperative visual control. These approaches have demonstrated exceptional tolerability [196]. Given the substantial therapeutic benefits associated with focal nanothermic action in tumor treatments, magnetite nanoparticle-based therapy represents a highly promising option in this field [197]. NanoTherm^®^ therapy, recognized as the world’s first MNP-based intervention for prostate and brain tumors, has undergone extensive preclinical evaluation and has progressed to clinical assessments [198]. Clinical trials have also been conducted to investigate MNPs in MH therapy for prostate cancer and glioblastoma [198].

MRI is recognized as one of the most advanced non-invasive techniques employed in clinical environments. The technique uses protons found in the human body. When exposed to a radiofrequency pulse, these protons become excited and realign with the magnetic field, allowing for tissue imaging. MNPs have been approved for use as contrast agents within clinical practice. Ferucarbotran/Resovist^®^ is a medicinal product designed for intravenous administration as an MRI contrast agent for the assessment of liver and spleen lesions [199,200]. It is available in syringe formulations of 0.9 mL and 1.4 mL and comprises carboxydextran-coated multi-core iron oxide nanoparticles. Ferumoxtran-10/Combidex^®^ (ultrasmall dextran-coated MNPs) is employed in MRI for the evaluation of the reticuloendothelial system [201,202]. Additionally, Ferumoxil/Lumiren^®^ (siloxane-coated MNPs) and ferristene/Abdoscan^®^ (ultrasmall MNPs coated with polystyrene(sulfonated styrene-divinylbenzene copolymer) are used for bowel imaging [203,204].

Ferumoxytol/Feraheme^®^ is a pharmaceutical product developed by AMAG Pharmaceuticals, based in Waltham, MA, USA. This product is intended for intravenous administration specifically for the treatment of iron deficiency anemia in adult patients diagnosed with chronic kidney disease [205,206]. Feraheme^®^ consists of carbohydrate-coated iron oxide nanoparticles and is provided in 17 mL vials, containing a concentration of 30 mg of iron (Fe) per mL.

A significant potential for MNPs is their use in magneto-responsive drug delivery systems for clinical applications. In the mid-1990s, the inaugural preclinical study investigating MNPs as delivery vehicles was conducted using human colon and renal cancer tumor-bearing murine models [207]. During this investigation, MNPs noncovalently loaded with epirubicin (4′-epidoxorubicin) were administered intravenously. The treatment resulted in complete tumor remission in the animal subjects. Subsequently, a Phase I clinical trial demonstrated successful drug accumulation at the target site in approximately 50% of patients (7 out of 14) with advanced solid tumors that had previously undergone unsuccessful treatment. Other experiments successfully demonstrated the effective delivery of mitoxantrone bound to starch-coated ultrasmall superparamagnetic iron oxide cores [208]. This method was applied to VX2-induced rabbit carcinoma and proved effective in eliminating tumors following approximately 35 days of intra-arterial administration.

The prevailing consensus derived from a range of preclinical and clinical trials suggests that MNPs exhibit minimal to no cytotoxic activity when administered at concentrations up to 100 μg Fe/mL, and potentially up to 8 mg Fe/mL in specific formulations, such as ferumoxytol [209]. Clinical dosing for these MNPs in human patients is typically established between 0.56 and 8 mg Fe/kg of body weight, which is considerably lower than the normal blood iron concentration, approximately 33 mg Fe/kg of body weight, and relatively modest in comparison to total body iron, estimated at approximately 3500 mg [210]. Nevertheless, despite the low doses, side effects and allergic reactions have been reported. It is posited that the observed toxicity associated with these nanoparticles is not primarily attributable to the magnetite cores but rather to factors including particle size, surface coatings, and stability in biological media or serum [110]. Furthermore, it is imperative to recognize that non-colloidal MNPs that lack stability in aqueous solutions and tend to precipitate may pose toxic risks to cellular and tissue viability. Given the potential for even minor alterations in MNP formulation to elicit significant changes in cytotoxic behavior, it is advisable to conduct a thorough evaluation of the toxicity associated with each unique MNP configuration prior to advancing to clinical trials.

## 6. Conclusions and Future Perspectives

The use of magnetic iron oxide nanoparticles in drug delivery and theranostic applications has garnered significant attention in recent years. Thus, it is imperative to diligently evaluate the potential toxicity of the nanoparticles intended for medical purposes. Nanotoxicity is influenced by various factors, including the dimensions and morphology of the nanoparticles, their surface chemistry, and charge, as well as their interactions with components of blood serum, biodistribution, and clearance within the biological system. Technical parameters such as dosage, exposure duration, frequency of administration, and the precursors utilized in nanoparticle preparation also substantially influence toxicity. Moreover, nanoparticles with similar properties may exhibit varying toxicity in disparate experimental models. An in-depth understanding of molecular-level interactions is pivotal in elucidating nanoparticle toxicity towards specific tissues and organs. The primary mechanisms and factors contributing to nanotoxicity encompass oxidative stress, interactions with genetic material, dysregulation of gene expression, cell membrane disruption, alterations in the cell cycle, inflammatory responses, disturbances in iron homeostasis, and cellular motility.

Since nanotoxicity is influenced by a multitude of factors, each type of designed nanoparticle should undergo thorough and separate testing. Typically, initial toxicity tests are conducted in vitro. Establishing an in vitro model for toxicity study is imperative for determining appropriate dosage and concentration levels for further evaluation in an in vivo system [21]. Consequently, in vivo studies are essential for discerning the actual and final nanotoxicity impact on the body.

Magnetic nanoparticles constitute a burgeoning study area with extensive potential in numerous medical applications. The imperative for precise targeting, coupled with the escalating challenge of drug resistance in treating specific cancerous tissues, underscores the necessity for targeted approaches employing MNPs. Significantly, MNPs can selectively impact healthy and cancerous cells, precipitating the termination of solely the cancerous cells, which is a highly desirable attribute in cancer treatment. Nanoparticles can be functionalized with various specific molecules, such as antibodies, which are carefully chosen to selectively interact with target receptors on the surface of particular cells. Furthermore, MNPs can be guided to specific sites within the body by applying an external magnetic field, thereby reducing potential side effects. Additionally, the hyperthermic effect elicited by MNPs can be applied in tandem with chemotherapy, thereby increasing the overall treatment efficacy. Considering all of the aforementioned factors, magnetic nanoparticles present as highly promising systems for pharmaceutical delivery and other medical applications. Therefore, thorough evaluations must be carried out to fully understand and leverage their potential.

## Figures and Tables

**Table 1:** The summary of the toxicity of diverse MNPs toward different models.

Coating	Size (TEM/D_H_)	Model	Dose	Significant Methods for Toxicity Assessing	Toxicity	Ref
PEG ^1^ 2000 Ethylene glycol	34 nm/ 325 nm 270 nm/ 1100 nm	D407, A548, MV35 and B12F10 cells	0.05–0.2 mg/mL	MTT assay	No significant toxicity was observed.	[162]
Bare	10–50 nm (AFM)/ 50 nm	Mouse embryonic fibroblasts NIH3T3	32.5 ng/mL	XTT assay	No significant toxicity was observed.	[163]
PA-PEG phosphonic acid HA-PEG hydroxamic	12 nm/ 40 nm (PA) 12 nm/ 72 nm (HA)	Primary human peripheral leucocytes	0.12–75 µg/cm	Measurement of ^3^H-thymidine incorporation into DNA of cells	No significant cytotoxic effect of PA-PEG@MNPs and HA-PEG@MNPs was found after 24 and 72 h of incubation.	[164]
Human-like collagen (HLC) protein	8,17,24 nm/32.2, 51.8, 84.4 nm	BHK-21 cells	100 μL of 12.5–100 μg/mL	WST-8 assay using Cell Counting Kit-8 (CCK-8)	No toxicity was observed for all concentration ranges and sizes, regardless of their incubation time.	[165]
ε-Poly (L-lysine) carbon dots	2–5 nm/-	Mouse MC3T3-E1 cells Human red blood cells	0.1, 0.5, 1 mg/mL	CCK-8 kit (Cell counting kit-8) assay Homolysis	No toxicity was observed. Low concentrations of MNPs (0.1 mg/mL) possessed acceptable hemocompatibility.	[166]
PEG-Arginine	18 nm/ 230 nm	HFF2 and HEK293 cells Human red blood cells	0.06–0.40 mg/mL 10 mg/mL	MTT assay Hemolysis assay	No significant cytotoxicity after exposure to any MNPs was observed. MNPs did not affect HRBCs of the blood.	[167]
Curcumin-PEG	24.33–34.24 nm/-	MCF7 cells Human red blood cells	1–100 µg/mL 10 mg/mL	MTT assay Hemolysis assay	MNPs did not show any toxicity. Non-hemolytic response was observed.	[168]
PAA ^2^-CF ^3^	9.2 nm/-	Splenic cells from rat Albino mice	7.8–1000 μg/mL Single dose of 100 μg/mL PAA@CF-MNPs	Trypan blue dye exclusion method and MTT assay Blood biochemistry	Non-significant cell growth stimulatory effects were observed at 1000, 62.5, 15.6, and 7.8 μg/mL and non-significant cell growth inhibitory effects at 500, 250, 125, and 31.25 μg/mL. The levels of ALT and AST showed a non-significant increase over the usual control group. The renal function parameters (serum urea and creatinine) were normal.	[169]
PAA-co-3-DEAPA ^4^	4.5 nm/2–8 nm	HUVEC cells Mice	Not available 0.2 mL of 0.138 mM solution	MTT assay Histology	No toxicity was observed. No abnormal changes were observed.	[170]
Ag/Fe_3_O_4_-CS ^5^-PVA ^6^/Ag	Not available	HEK293 and LO2 cells Mice	1.25–40 μg/mL Not available	CCK-8 kit (Cell counting kit-8) assay Hemolysis Histology	No apparent toxic effects on cells were observed. Slight hemolysis was observed. No abnormal changes were observed.	[171]
Chitosan	-/320 nm	ECs from Wistar rats Human blood F1 mice	1–100 µg/mL 1, 10 and 100 µg/mL 30 mg/kg	MTT assay NO production The erythrocyte sedimentation rate (ESR) and hematology Histology	No viability inhibition of cells was observed. The presence of the MNPs did not affect basal NO production. No significant differences in ESR in comparison to controls were observed. The hemolytic effect was not observed with any of the tested doses assayed. Histological examinations of the liver, stomach, intestine, lungs, and brain showed no changes at the end of the sub-acute exposure to MNPs in any of the mice after 28 days. The kidneys exhibited granular interstitial tissue, which was compatible with periarteriolar interstitial nephritis compared to the control. In the spleen, an increase in the presence of megakaryocytes was observed.	[172]
Oleic acid	10, 20, 30, 40 nm/14, 25, 34, 43 nm	Kunming mice	20 mg/kg	Blood biochemistry	The critical hepatic indicators were not significantly altered independent of the sizes of MNPs treated compared with the control. The kidney function indicators exhibited levels similar to those of the control group.	[173]
Rhodamine B isothiocyanate (RITC) within a silica shell	-/50 nm	ICR mice	100, 50, 25 mg/kg	Blood biochemistry, hematology and histology Neurotoxicity assays	No significant changes were observed in the histological, hematological, and biochemistry tests. MNPs could penetrate the BBB without altering its function.	[104]
DOX ^7^- 4,4′-Azobis (4-cyanovaleric acid)	212 nm/-	ICR mice	200 μL of solution of free DOX and DOX-loaded MNPs at a dose normalized to be 3 mg/kg DOX equiv.	Histology	MNP-Azo-DOX did not produce histopathological signs of cardiotoxicity like that observed for free DOX.	[174]
PEI ^8^ PAH ^9^ PDADMAC ^10^	-/147 nm -/116.5 nm -/139 nm	Human lung carcinoma cell line (A549).	100 µg/mL	MTT assay Resazurin reduction assay	The MTT test showed a slight decrease in the activity of cytosolic hydrogenases in all variants, most pronounced in the variant with MNPs-PEI. A test with resazurin reduction showed that incubation with MNP-PEI slightly stimulated mitochondrial enzymes.	[175]
BSA ^11^ BSA BSA-PEG	-/80 nm -/40 nm -/40 nm	Human fibroblast cells Human glioblastoma U251 cells	10^−3^–10^−7^ M	MTT assay LDH assay Intracellular oxidative activity evaluated by a dichloro-dihydrofluorescein diacetate (DCFHDA) fluorescent dye. Comet assay	After 48 h, the highest concentration of BSA-IONP-80 and BSA-IONP-40 showed some cytotoxic effect, which was more robust in the case of BSA-MNP-40. BSA-MNP-PEG toxicity was almost negligible in comparison to other types of MNPs. No significant change in the confluency area of U251 cells was observed. The measurements of LDH activity after 24 h of incubation with MNPs have not shown any differences in cell membrane integrity for all samples in all concentrations tested. For 24 h, all types of MNPs provided less ROS production than positive control. After 48 h of HF-cell incubation, a noticeable increase in fluorescence level was observed. Signal intensity was almost equal to fluorescence intensity related to cells treated with a control solution of H_2_O_2_. As for U251 cells, a significantly lower fluorescence level was observed compared with the control solution of H_2_O_2_. 24 h after incubation with different types of synthesized MNPs, there was no difference in DNA damage level between the control and experimental nanoparticle groups. An increase in DNA fragments was detected after 48 h of HF-cell incubation with BSA-MNP-40. In the case of the U251 cell line, no significant difference between DNA fragmentation in control and treated cells for all types and concentrations of MNPs used was observed.	[19]
PEG	3 nm/21 nm 14–20 nm/56 nm	Mouse microglia cell line N13 Zebrafish embryos	0.1–100 μg/mL 0.01–100 μg/mL	MTT assay Evaluation of the hatching and survival rates of zebrafish embryos	No significant cytotoxicity after 24 h of exposure to any MNPs was observed. Higher concentrations of MNPs (10 μg/mL and 100 μg/mL) showed an increased hatching rate compared to control non-exposed embryos. No mortality or malformations were observed in the embryos exposed to different doses of particles at 48 h.	[176]
Dextran	10–20 nm/40–160 nm	L929 fibroblast Albino rats (Wistar), Albino guinea pigs (Hartley), and Albino mice (Swiss)	100–800 μg/mL 300–2000 mg/kg	MTT assay Blood biochemistry and hematology Lymphocyte proliferation assay Detection of 8-OHdG by ELISA Mammalian bone marrow chromosomal aberration study	No proliferation inhibition in the whole range of MNP concentrations was observed. The biochemical and hematological assessments following oral administration of MNPs were not significantly different from those in the control group. Seven days after exposure, a slight increase in cell number was observed in both T and B lymphocytes compared to the control. After 14 days and 21 days, the proliferation of T and B cells was reduced compared to day 0. The levels of 8-OHdG in mitochondrial DNA of MNP-exposed groups were comparable with those of control values. MNPs did not significantly affect the chromosome aberration frequencies in bone marrow cells or cell mitotic indices.	[42]
Bare APTMS ^12^ TEOS ^13^/APTMS	-/10 nm -/100 nm -/150 nm	HDFs and HT-1080 cells	200–1000 μg/mL	Cell Counting Kit-8 (CCK-8) assay Comet Assay	A slight toxicity was observed in HDFs treated with increasing concentrations of each MNP in a dose-dependent manner. MNPs modified with APTMS resulted in significant dose-dependent genotoxicity against normal cells. Bare and TEOS/APTMS-coated MNPs resulted in neither extensive nor dose-dependent damage to the DNA stability in both cells.	[20]
Oleic acid-chitosan (N1) Oleic acid-chitosan and glutaraldehyde as cross-linker (N2)	10 nm/ 369 nm (N1) 10 nm/ 238 nm (N2)	ECs cultures from Wistar rats Mice	1, 10, 100 μg/mL 30 mg/kg	MTT assay Measurement of NO production	ECs treated with N1 nanoparticles for 6–24 h compared to control cells showed maximal cell viability. In contrast, a significant reduction in cell viability was evidenced in the treatment with the highest dose (100 μg/mL) after 36 h. The treatment with N2 MNPs did not affect cell viability in the whole range of doses and times explored. Endothelial NO production was not affected by the exposure to N1 or N2.	[177]
DMSA ^14^	-/60 nm	OLN-93 cells	0.25, 1, 4 mM	LDH assay Staining with PI, H33342, and rhodamine 123 dyes	No significant increase in the extracellular activity of the enzyme LDH was observed. Cultures incubated with 0.25 or 1 mM MNPs hardly contained any PI-positive cells despite the presence of many cells, which was demonstrated by H33342 staining. Exposure to 0.25 mM MNPs did not increase ROS production, while many rhodamine 123-positive cells were present in cultures exposed to 1 or 4 mM MNPs.	[178]
Bare Chitosan	6 nm/- 8 nm/-	HeLa, A549 and HeK293 cells	0.5, 2, 4 μg/μL	MTT assay and AO/EB staining	The toxic effect of chitosan-MNPs on A549 and HeLa cells was moderate compared to bare MNP treatment, and this toxicity was found to be time- and dose-dependent. In the case of Hek293 cells, bare MNPs led to toxic effects, whereas chitosan-coated MNPs did not cause any significant toxicity. Chitosan-MNPs caused less apoptosis in healthy and cancer cell lines than bare MNPs.	[18]
Carboxymethyl chitosan	46–57 nm/-	MCF7 human breast cancer cells and 3T3 fibroblasts	6.25–100 μg/mL	MTT assay	MNPs displayed toxic effects against MCF-7 cells. No toxicity towards 3T3 fibroblasts was observed.	[179]
Bare Silica-APTES ^15^	12 nm/- 26 nm/-	HeLa and A549 cells	0.5, 1, 2.5, 5 nM	WST-8 assay DCF fluorescence as a reporter of ROS generation	Bare MNPs showed a substantial viability reduction at high concentrations (2.5, 5 nM) in both cell lines, whereas coated MNPs showed no sign of toxicity. A significant ROS generation was observed in cells treated with bare MNPs. Coated MNPs induced low levels of ROS.	[17]
Curcumin	9.9 nm/406 nm	HUVECs cells	200 µL of medium at concentrations 1–1000 μg/mL	A calcein AM red-orange viability assay	Curcumin-coated MNPs showed less cell death relative to uncoated MNPs at variable concentrations.	[180]
PEG PEG PEI	10 nm/ 16.5 nm 30 nm/ 38.5 nm 10 nm/ 17.2 nm	RAW264.7 macrophage, SKOV-3 cancer cells BALB/c mice	3.125–100 µg/mL 1.5–5 mg/kg	MTS assay Hematology and blood biochemistry Histology	SEI-10 induced dose-dependent cytotoxicity against both RAW264.7 macrophages and SKOV-3 cancer cells at the test concentrations, and SKOV-3 cells were relatively more susceptible to SEI-10 toxicity than RAW264.7 macrophages. No appreciable cytotoxic effects were observed for SMG-10 and SMG-30 at 25 µg/mL; slight cytotoxicity was shown above 50 µg/mL. The hematology and blood chemistry results on day seven post-injection showed that AST, total bilirubin, BUN, and creatinine were within the normal range, except that the level of ALT enzyme in mice treated with SMG-10 slightly increased compared to the PBS control. On day 14 post-injection, the increased ALT level in SMG-10-treated mice returned to normal. In mice treated with SMG-10 and SMG-30, slight mononuclear cell infiltration in the portal area of the liver was identified. Splenic plasmacytosis was noted in mice treated with SMG-30.	[181]
Curcumin/Alginate	12–15 nm/98 nm	Sarcoma 180 cancer cells Mice	0.01–1000 µg/mL 80–120 mg/kg	MTT assay Blood biochemistry Histology	Cytotoxicity was observed only at high doses. Biochemical assay data indicated that AST and ALT values were higher in the treated mice than in the control mice, with a significant difference in AST values. In contrast, the levels of BUN and creatinine did not change significantly. The histological structures of livers changed compared to those in the control group, with the appearance of vacuolated hepatocytes.	[182]
L-glutathione	-/60 nm	*Caenorhabditis elegans*—non-parasitic nematodes	10–200 mg/L	Mortality Growth Locomotion Fertility	The presence of nanomaterial increased mortality without a specific relationship between the concentration and the number of dead nematodes. A slight decrease in the length of the worms was observed for control. The decrease in locomotion was not significant. A decrease in the number of eggs placed was observed for each nematode by increasing the nanomaterial concentration in the medium.	[183]
Bare MNPs pPEG-AC ^16^-poly(amidoamine-paraben)-PEG	9 nm/11.68 nm Nano-clusters of 100–150 nm/-	Swiss albino mice	5, 10, and 25 mg/kg	Blood biochemistry Histology	The highest dose of bare MNPs induced significant malfunctions in systemic biomarkers. In contrast, lower doses (5 and 10 mg/kg) of uncoated and all coated MNPs did not alter these biomarkers. All tissue sections, including liver, kidney, spleen, and heart, excluding lungs, treated with the highest dose (25 mg/kg) of bare MNPs demonstrated significant iron deposition. Lower doses (5 and 10 mg/kg) of uncoated MNPs and all coated NPs showed no iron accumulation.	[15]
L-carnosine	-/120 nm	BALB/c mice	Equivalent carnosine dose of 200 mg/kg/day	Blood biochemistry Histology	A significant increase in the liver enzymes (ALT and AST) was observed. Iron accumulations were detected. No structural or histopathological changes were observed in the liver tissues, indicating no tissue damage.	[184]

^1^ PEG—poly(ethylene glycol); ^2^ PAA—poly(acrylic acid); ^3^ CF—cobalt ferrite; ^4^ DEAPA—3-(diethylamino)-propyl amine; ^5^ CS—chitosan; ^6^ PVA polyvinyl alcohol; ^7^ DOX—doxorubicin; ^8^ PEI—poly(ethylene imine); ^9^ PAH—poly(allylamine hydrochloride); ^10^ PDADMAC—poly(diaallyldimethylammonium chloride); ^11^ BSA—bovine serum albumin; ^12^ APTMS—(3-aminopropyl)trimethoxysilane; ^13^ TEOS—tetra-ethoxysilane; ^14^ DMSA—dimercaptosuccinic acid; ^15^ APTES—(3-aminopropyl)triethoxysilane; ^16^ AC—amincellulose.

## Abbreviations

AC	amino cellulose.
ACur	alternating current.
ALP	alkaline phosphatase.
ALT	alkaline aminotransferase.
AMF	alternating magnetic field.
ANT	adenine nucleotide translocase.
APTES	(3-aminopropyl)triethoxysilane.
APTMS	(3-aminopropyl)trimethoxysilane.
BBB	blood-brain barrier.
BCECs	brain capillary endothelial cells.
BSA	bovine serum albumin.
CAT	catalase.
CF	cobalt ferrite.
CMC	N-carboxymethyl chitosan.
CPK-MB	creatine phosphokinase-MB.
CS	chitosan.
CSF	cerebrospinal fluid.
DEAP	3-(diethylamino)-propyl amine.
DEX	dextran.
DMSA	dimercaptosuccinic acid.
DOX	doxorubicin.
EGFR	epidermal growth factor receptor.
ETC	electron transfer chain.
GGT	gamma-glutamyl.
GPx	glutathione peroxidase.
GSH	glutathione reductase.
HUVECs	human umbilical vein endothelial cells.
ICG	indocyanine green.
LDH	lactate dehydrogenase.
LPS	lipopolysaccharide.
MDA	malondialdehyde.
MNPs	magnetic nanoparticles.
MPTP	mitochondrial permeability transition pore.
MRI	magnetic resonance imaging.
MTD	magneto-thermodynamic.
MTT	3-(4,5-dimethylthiazol-2-yl)-2,5-diphenyltetrazolium bromide.
MTX	methotrexate.
NAC	N-acetylcysteine.
NSCLC	non-small cell lung cancer.
NSCs	neural stem cells.
PAA	polyacrylic acid.
PAH	poly(allylamine hydrochloride).
PAMAM	polyamidoamine.
PDADMAC	poly(diallyl dimethylammonium chloride).
PEG	poly(ethylene amine).
PEI	poly(ethylene imine).
PLL	poly-L-lysine.
PVA	polyvinyl alcohol.
ROS	reactive oxygen species.
SOD	superoxide dismutase.
TAC	tacrolimus.
TEOS	tetraethoxysilane.
TLR4	Toll-like receptor 4.
VAN	vancomycin.
VDAC	voltage-dependent anion channel.
